# Multidimensional burden of scarring alopecia in women: findings from the CAPAIR study

**DOI:** 10.1097/JW9.0000000000000268

**Published:** 2026-07-02

**Authors:** Jasmine Levine, Ross O’Hagan, Jordan Talia, Jean R. Pickford, Itisha S. Jefferson, Morinola Shobajo, Emma Guttman-Yassky, Benjamin Ungar

**Affiliations:** a Department of Dermatology, Icahn School of Medicine at Mount Sinai, New York, New York, USA; b Scarring Alopecia Foundation, Kulpsville, Pennsylvania, USA; c Loyola University Stritch School of Medicine, Maywood, Illinois, USA; d University of Minnesota Department of Dermatology, Minneapolis, Minnesota, USA

**Keywords:** central centrifugal cicatricial alopecia, frontal fibrosing alopecia, lichen planopilaris, patient-reported outcomes, primary cicatricial alopecia, quality of life

## Abstract

**Background:**

Primary cicatricial alopecias are rare inflammatory disorders that disproportionately affect women and can cause permanent hair loss along with painful scalp symptoms. While their impact on quality of life has been described, comprehensive patient-reported data remain limited.

**Objective:**

To characterize symptoms, lifestyle disruption, and financial burden among women with central centrifugal cicatricial alopecia, lichen planopilaris, and frontal fibrosing alopecia.

**Methods:**

A nationwide cross-sectional survey was distributed via the Scarring Alopecia Foundation. Analyses included female respondents with self-reported centrifugal cicatricial alopecia, lichen planopilaris, or frontal fibrosing alopecia, stratified by subtype, hair loss severity, and symptom burden.

**Results:**

Among 917 women, physical symptoms, lifestyle disruption, and disease-related spending were commonly reported. Burning and pain/tenderness were significantly associated with greater impairment in work productivity, daily activities, and treatment-related costs, while greater scalp involvement also corresponded to increased burden. However, this pattern did not hold among a small subset of completely bald respondents, suggesting that psychosocial burden increases with disease severity but may shift once a certain threshold is surpassed.

**Conclusion:**

Primary cicatricial alopecias impose substantial physical, psychosocial, and financial burden on women. These findings underscore the need for improved physician awareness and greater support for women managing this chronic, stigmatizing disease.

**What is known about this subject in regard to women and their families?**

Primary cicatricial alopecias, including central centrifugal cicatricial alopecia, lichen planopilaris, and frontal fibrosing alopecia, more commonly affect women and are associated with permanent hair loss, painful scalp symptoms, and significant psychosocial distress. Previous studies have documented impaired quality of life, but most have been limited by small sample sizes and the use of nonspecific questionaries that do not comprehensively reflect patient experiences.

**What is new from this article as messages for women and their families?**

This study presents the largest patient-reported survey to date focused on women with scarring alopecia. It provides detailed insight into symptom severity, lifestyle disruption, and financial impact. The findings highlight how certain symptoms and levels of hair loss are linked to greater burden and offer new opportunities to improve clinical care and patient support. The results also emphasize the need for increased clinician awareness and public understanding of these often-overlooked conditions.

## Introduction

Primary cicatricial alopecias (PCAs) are rare inflammatory scalp disorders defined by immune-mediated destruction of hair follicles, followed by fibrosis and potentially permanent hair loss.^1,2^ Although exact prevalence is difficult to determine because of population variability and methodological hurdles, estimates range from 3.7 to 7.2%.^3–5^ Central centrifugal cicatricial alopecia (CCCA), lichen planopilaris (LPP), and frontal fibrosing alopecia (FFA) are the most common PCA subtypes, each disproportionately affecting women but with distinct clinical features.^3,6,7^ CCCA primarily affects young to middle-aged Black women, typically beginning at the vertex with progressive hair loss.^5,6^ LPP and FFA are more common in mid-to-late adulthood among White women and present with patchy or diffuse alopecia, often accompanied by perifollicular erythema and scale.^8,9^ FFA is further distinguished by its predilection for the frontotemporal hairline, often with accompanying loss of eyebrows or other body hair.^10,11^

Despite differing presentations, all PCAs involve progressive hair loss, often accompanied by painful scalp symptoms.^8,9,12–14^ This combination can cause significant psychosocial distress, particularly given the potential for permanent scarring, which results from sustained inflammation over time, making early diagnosis and treatment critical to prognosis.^3,6,9,12^ Yet PCAs remain underdiagnosed and undertreated due to their clinical heterogeneity and limited recognition, even among dermatologists.^5–7,15^ Moreover, no Food and Drug Administration–approved treatments currently exist, available therapies often demonstrate limited efficacy and add to patient burden.^1,9,10,16,17^ This lack of therapeutic advancement reflects broader gaps in pathophysiologic understanding, with relatively few studies focused on elucidating disease mechanisms or informing the development of targeted interventions.^2,7,9,15,18,19^ As a result, clinicians continue to face substantial challenges in altering disease course and optimizing patient outcomes.^2,3,7,17^

PCAs, with accompanying physical and emotional burden, can profoundly disrupt daily life.^1,3,9^ Indeed, while the psychosocial effects of scarring alopecia have received increasing attention in recent years, few studies have comprehensively assessed patient-reported impact and outcomes across large and diverse cohorts.^3,9,17,20^ As a result, key aspects of how these chronic and stigmatizing disorders affect patients’ lives remain poorly understood.^3,14,17^ To address this gap, the Scarring Alopecia Foundation (SAF) conducted the nationwide Cicatricial Alopecia Patient Assessment and Impact Report (CAPAIR) survey. In this study, we analyzed CAPAIR responses related to symptoms, lifestyle disruption, and financial burden among individuals with PCA, with the aim of better characterizing overall disease burden and identifying clinical features associated with greater impact.

## Methods

### Study design

The CAPAIR study was a cross-sectional, deidentified, patient-reported survey distributed via email to the SAF membership listserv across 6 time points between December 3 and 16, 2022. Eligible participants were ≥18 years with a self-reported diagnosis of PCA. To improve interpretability and statistical power, analyses were limited to female respondents with the 3 most common subtypes, CCCA, LPP, and FFA. Responses from males and those reporting rarer subtypes were excluded because of small sample sizes and to reduce misclassification bias.

### Survey content

The survey captured data across multiple domains, including sociodemographic characteristics, PCA diagnosis, physical symptoms, hair loss severity, and the financial, lifestyle, and occupational impact of the disease. Physical symptoms were rated on a 4-point Likert scale (never, rarely, sometimes, always) to capture current symptom burden at the time of survey completion, and respondents estimated scalp hair loss using defined brackets: ≤10%, 11 to 25%, 26 to 50%, ≥51%, and bald. Financial burden was assessed based on self-reported expenses across 4 categories, with responses grouped into 6 spending brackets. Lifestyle and occupational burden were evaluated through several questions referencing the prior 7 days. Respondents also rated the extent to which health issues impaired their work productivity and daily functioning on a scale from 1 (no impairment) to 10 (complete impairment).

### Statistical analysis

Descriptive statistics were used to summarize sociodemographic characteristics and survey responses. Respondents with CCCA were compared to those with LPP or FFA, which were grouped because of frequent diagnostic overlap and common classification of FFA as a clinical variant of LPP. Additional stratification by percentage of scalp involvement assessed whether hair loss severity predicted burden. To evaluate symptom-specific predictors, outcomes were stratified by severity of individual symptoms, dichotomized as “less severe” (never/rarely) or “more severe” (sometimes/always) based on Likert ratings. Comparative analyses used Pearson’s *χ*^2^ and Kruskal–Wallis rank-sum tests; analyses were performed using R.

## Results

### Distribution

The CAPAIR survey was successfully delivered to 6,374 individuals on its initial launch. Of these, 2,768 recipients opened the message (43% open rate), and 444 clicked on the survey link (7% click rate). Across all 6 SAF listserv distributions between December 3 and 16, 2022, open rates ranged from 41 to 49%. A total of 1,048 individuals ultimately completed the survey, corresponding to a response rate of 16% among those who successfully received the initial email and 38% among those who opened it.

### Sociodemographic information

A total of 917 female respondents with self-reported diagnoses of CCCA, LPP, or FFA met the inclusion criteria for analysis. The mean age was 59 (SD: 14). Most identified as White (76%) or Black (22%), and 64% held a bachelor’s degree or higher. A majority reported household incomes over $100,000/year and were married (65%), with 13% divorced and 14% never married. Of the total, 196 (21%) reported CCCA, and 721 (79%) reported LPP/FFA. The CCCA subgroup was younger (mean age: 51) and predominantly Black (91%), while the LPP/FFA subgroup was older (mean age: 62) and mostly White (95%) (Table 1).

**Table 1.** Sociodemographic characteristics of survey respondents, stratified by subtype of primary cicatricial alopecia, including central centrifugal cicatricial alopecia, lichen planopilaris, and frontal fibrosing alopecia

Characteristic	N^a^	CCCA, N (%)	LPP/FFA, N (%)	Overall, N (%)
Age (SD)	864	51 (13)	62 (14)	59 (14)
Gender	917			
Female		196 (100%)	721 (100%)	917 (100%)
Race	917			
American Indian or Alaska Native		0 (0%)	1 (0.1%)	1 (0.1%)
Asian		0 (0%)	8 (1.1%)	8 (0.9%)
Black or African American		172 (91%)	27 (3.9%)	199 (22%)
White		17 (9.0%)	661 (95%)	678 (76%)
Other/unknown		7	24	31
Education	605			
High school or less		4 (2.9%)	35 (7.5%)	39 (6.4%)
Some college/associate’s		44 (32%)	135 (29%)	179 (30%)
Bachelor’s degree or higher		89 (65%)	298 (64%)	387 (64%)
Annual household income	740			
Lower-middle income (<100k)		81 (47%)	247 (44%)	328 (44%)
Upper-middle income (100-250k)		76 (44%)	238 (42%)	314 (42%)
High income (250k+)		15 (8.7%)	83 (15%)	98 (13%)
Marital status	903			
Married		94 (49%)	496 (70%)	590 (65%)
Unmarried		97 (51%)	216 (30%)	313 (35%)

CCCA, central centrifugal cicatricial alopecia; FFA, frontal fibrosing alopecia; LPP, lichen planopilaris; SD, standard deviation.

a Sample sizes may vary because of unreported data, and percentages are calculated based on the complete data of the column.

### Physical symptoms

Among 844 respondents, 70% reported at least 1 physical symptom at any frequency (rarely, sometimes, or always), including 86% of those with CCCA and 73% with LPP/FFA. The frequency distribution of current symptoms differed significantly by PCA subtype (*P* < .001). Itching was most common (80% CCCA and 84% LPP/FFA), followed by pain/tenderness (68% CCCA and 66% LPP/FFA) and burning (42% CCCA and 47% LPP/FFA), with no significant differences by subtype. Dryness/crusting/scaling was more frequent in LPP/FFA (55%) than CCCA (35%), with a significant difference in frequency distribution (*P* < .001), while blistering was rare overall (16%) and did not differ by subtype. Hair-related symptoms were nearly universal: 92% of CCCA and 97% of LPP/FFA respondents reported hair thinning, with over half in each group rating it as “always” (52% CCCA and 66% LPP/FFA), and a significant difference in frequency distribution by subtype (*P* = .004). Eyebrow thinning was significantly different (*P* < .001) and substantially more common in LPP/FFA (76 vs. 35% in CCCA) (Table 2).

**Table 2.** Survey responses on self-reported physical symptom burden, stratified by subtype of primary cicatricial alopecia, including central centrifugal cicatricial alopecia, lichen planopilaris, and frontal fibrosing alopecia

Characteristic	N^a^	CCCA, N (%)	LPP/FFA, N (%)	*P*-value^b^	Overall, N (%)
Current symptoms	844			<.001	
Never/rarely		46 (27%)	298 (44%)		344 (41%)
Sometimes/always		127 (73%)	373 (56%)		500 (59%)
Blistering	910			.828	
Never/rarely		183 (94%)	670 (94%)		853 (94%)
Sometimes/always		11 (6%)	46 (6%)		57 (6%)
Burning	844			.150	
Never/rarely		143 (77%)	472 (72%)		615 (73%)
Sometimes/always		42 (23%)	187 (28%)		229 (27%)
Dryness, crusting, scales	824			.006	
Never/rarely		143 (79%)	434 (68%)		577 (70%)
Sometimes/always		39 (21%)	208 (32%)		247 (30%)
Itching	719			.971	
Never/rarely		59 (37%)	203 (36%)		262 (36%)
Sometimes/always		101 (63%)	356 (64%)		457 (64%)
Pain/tenderness	804			.632	
Never/rarely		95 (54%)	354 (56%)		449 (56%)
Sometimes/always		81 (46%)	274 (44%)		355 (44%)
Thinning eyebrows	821			<.001	
Never/rarely		138 (75%)	221 (35%)		359 (44%)
Sometimes/always		47 (25%)	415 (65%)		462 (56%)
Thinning hair	732			.057	
Never/rarely		26 (17%)	61 (11%)		87 (12%)
Sometimes/always		131 (83%)	514 (89%)		645 (88%)

CCCA, central centrifugal cicatricial alopecia; FFA, frontal fibrosing alopecia; LPP, lichen planopilaris.

a Sample sizes may vary because of unreported data, and percentages are calculated based on the complete data of the column.

b Pearson’s *χ*^2^ test.

### Lifestyle

Among questions from the Work Productivity and Activity Impairment Questions, (WPAI), in the 7 days before completing the survey, 16% of respondents in both groups reported missing at least 1 hour of work because of their disease, while only 35% of the CCCA cohort and 50% of the LPP/FFA cohort reported working at all that week (excluding the percentage who reported 0 hours worked, this rises to approximately 36% of individuals missing work because of their disease). In the same timeframe, 22% of CCCA and 20% of LPP/FFA respondents reported taking time off for leisure, similar in proportion to missed work because of disease. Work productivity impairment was also prevalent, reported by 39% of respondents, with 13% rating it as ≥4/10. When limited to those employed, 45% reported impairment, including 15% with scores ≥4/10. Regular activity impairment similarly occurred, affecting 43% of respondents, with 15% rating it as ≥4/10 (Supplementary Table S1, https://links.lww.com/IJWD/A91).

### Finances

Most respondents reported ongoing monthly treatment costs, including 87% of those with CCCA and 90% with LPP/FFA. Of these, 37% in each group spent over $100/month, with a small subset exceeding $1,000. Nonprescription “silver bullet” treatments (defined as fads, trends, or alternative treatments) were also common: 66% of CCCA and 62% of LPP/FFA respondents reported spending over $100/year, with 16% of CCCA and 24% of LPP/FFA respondents exceeding $1,000/year, and 8% of the total cohort reporting expenditures of $2,501 to $5,000+. Travel-related costs to access specialized care were reported by 36% of CCCA and 34% of LPP/FFA participants, including over 6% in each group who spent more than $1,000/year. Concealment-related expenses (eg, wigs, toppers) were nearly universal, reported by 86% of CCCA and 69% of LPP/FFA respondents, with 18% in each group spending more than $1,000/year (Supplementary Table S1, https://links.lww.com/IJWD/A91).

### Predictors of burden

**Fig. 1.**
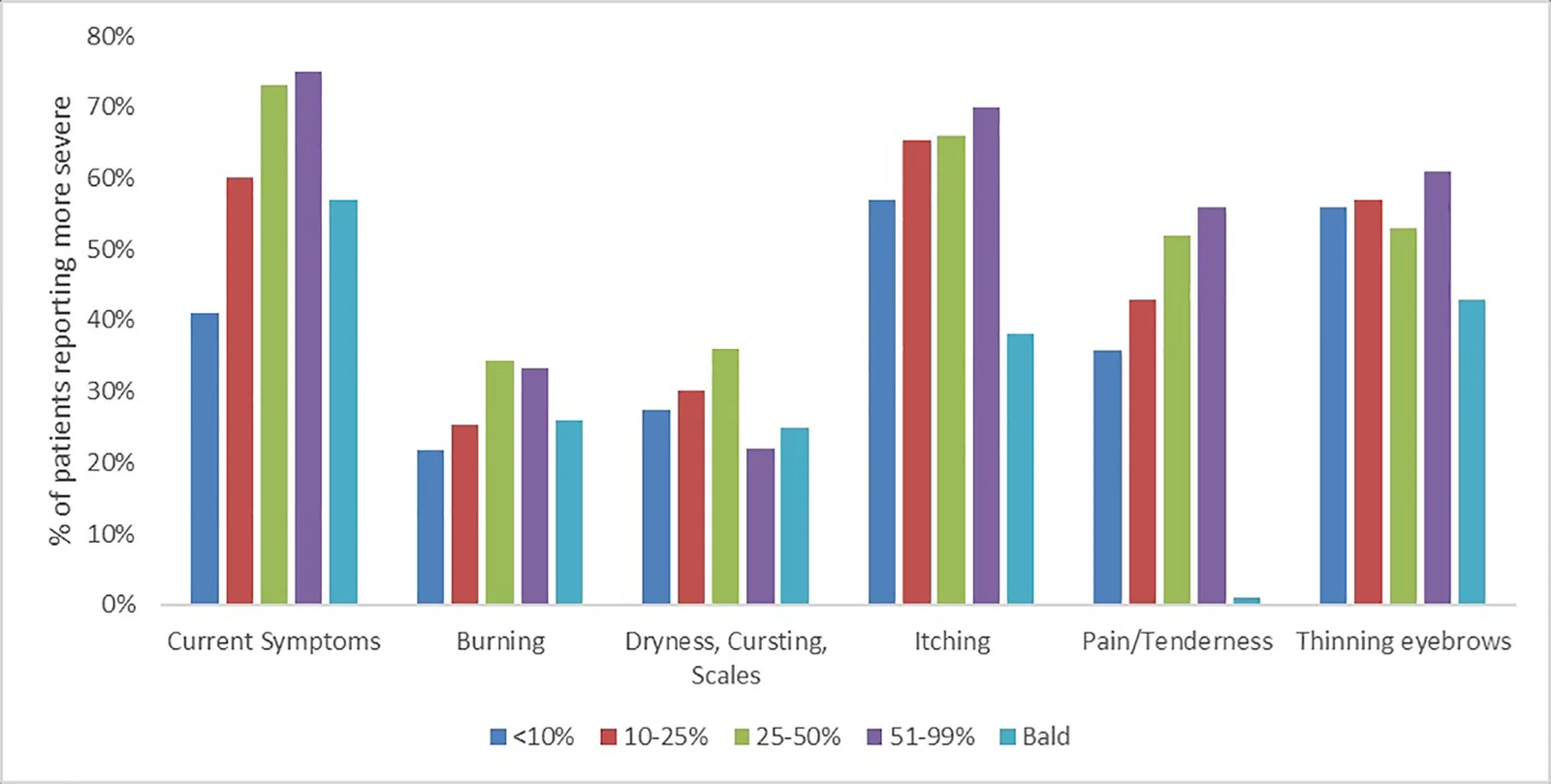
Bar graph demonstrating self-reported physical symptom severity, stratified by extent of scalp hair loss. Bars represent the percentage of respondents rating each symptom as “sometimes” or “always” present within each hair loss category.

Hair loss severity, defined by percentage of scalp involvement, generally corresponded with increased patient-reported burden (Supplementary Table S2, https://links.lww.com/IJWD/A92). Physical symptoms were reported by 54% of those with <10% involvement and 86% of those with ≥51%. Burning increased from 36% in the <10% group to 62% among those who were bald, with similar patterns observed for itching, pain/tenderness, and dryness/crusting/scaling. Eyebrow thinning was relatively consistent across groups but peaked among participants with ≥51% scalp involvement (Fig. 1). Lifestyle and financial burden also increased with hair loss severity, with greater impairment, productivity loss, and spending on concealment and nonprescription treatments reported in the 25 to 50% and ≥51% groups. However, responses from completely bald participants (n = 8) deviated from this pattern, indicating lower burden across several domains.

Among physical symptoms, burning and pain/tenderness showed the strongest associations with overall burden. Respondents reporting more severe burning had significantly higher mean work productivity impairment (1.73 vs. 0.97, *P* = .004), daily activity impairment (1.94 vs. 1.24; *P* = .024), monthly treatment costs (*P* = .008), and care-related travel expenses (*P* = .041) (Supplementary Table S3, https://links.lww.com/IJWD/A93). Similarly, those with more severe pain/tenderness reported significantly higher mean work productivity impairment (1.41 vs. 0.86; *P* = .003), monthly treatment costs (*P* = .034), and care-related travel expenses (*P* = .047), with a trend toward increased daily activity impairment (*P* = .068) (Supplementary Table S4, https://links.lww.com/IJWD/A94).

## Discussion

This large cross-sectional survey provides the most comprehensive characterization to date of patient-reported experiences with PCAs, capturing multidimensional burden across symptoms, functioning, and finances in over 900 women with CCCA, LPP, or FFA. Physical symptoms were prevalent throughout, and work disruption emerged as a prominent finding. Although only 46% reported working in the prior week (for reasons not captured, though disease-related impairment may have contributed), 36% of these employed respondents reported missing work because of their condition, and 45% reported impaired work productivity. Moreover, stratified analyses identified several clinical features linked to disproportionate burden. Burning and pain/tenderness were significantly associated with greater impairment in daily functioning, work productivity, and spending. Greater hair loss severity similarly corresponded to increased symptoms, spending, and lifestyle disruption. These findings help clarify which patients may face a heightened quality of life (QoL) burden and highlight opportunities for more targeted intervention.

Alopecia has long been recognized as a source of psychological distress, identity disruption, and impaired QoL, particularly among women.^3,21–23^ While this burden is well-documented, most studies have focused on nonscarring alopecias such as androgenetic alopecia and alopecia areata, with limited attention to PCAs.^3,9,22,24–26^ Yet PCAs differ meaningfully because of features that uniquely shape patient experience, such as irreversible scarring, chronic inflammation, and limited therapeutic options.^5–7^ While qualitative studies frequently describe profound psychosocial impact, quantitative research may underestimate this burden because of small sample sizes, variability in study design, and reliance on generic QoL questionnaires that lack sensitivity to disease-specific concerns, as highlighted in a recent systematic review.^3,17,27^ Several individual PCA cohorts have likewise shown only modest average QoL impairment when using generic instruments such as the Dermatology Life Quality Index (DLQI), with Chiang et al.^1^ even reporting lower DLQI scores than those previously observed in psoriasis or atopic dermatitis patients.^1,28,29^

Several studies have further used these generic instruments to examine clinical predictors of QoL impairment in PCA, but findings remain inconsistent. Among 50 CCCA patients, Agner et al. found that patient-reported rather than physician-assessed hair loss severity predicted impairment and overall QoL.^30^ The LPP Activity Index (LPPAI), a standardized composite measure integrating patient-reported symptoms with clinical signs, has been applied across multiple QoL studies with mixed results.^8,18^ Higher LPP Activity Index scores and greater hair loss severity were associated with poorer QoL in some cohorts,^9,31^ while other studies, such as those by Doche et al.^32^ and Chiang et al.^1^ have failed to replicate these associations, though Chiang et al.^1^ did note that >25% scalp involvement was independently associated with worse QoL. In FFA, Saceda-Corralo et al.^28^ reported that scalp discomfort, but not alopecia severity, was significantly associated with worse DLQI scores. Meanwhile, standardized metrics of severity such as the FFA Severity Index have shown limited alignment with reported QoL impairment.^21,33^

Studies have further attempted to use hair-specific instruments to better assess the psychosocial burden of PCAs, including the Women’s Androgenetic Alopecia QoL Questionnaire and Scalpdex (originally developed for seborrheic dermatitis and psoriasis), though both demonstrated limited correlation with LPP severity or patient experience.^14,34^ To further address these limitations, several tools have been developed specifically for PCAs. The FFA QoL Index, validated in 101 women with FFA, identified moderate-to-severe impairment in over one-third of patients and demonstrated stronger correlations with disease severity than DLQI.^23^ Likewise, the CCCA QoL Index revealed significant QoL impairment across multiple CCCA cohorts,^35–37^ with 1 study further associating younger age, longer disease duration, and frequent wig use with poorer CCCA QoL Index scores.^38^

Overall, the fragmented nature and inconsistent findings of prior research have hindered a comprehensive understanding of patient burden in PCA. Although subtype-specific QoL instruments may improve measurement sensitivity, developing separate tools for each disease is inefficient and may still fail to capture patients’ broader lived experience.^3^ This study helps address these limitations by surveying a large, diverse population and identifying clinically relevant predictors of burden, including physical symptoms and hair loss. Moreover, while physical and psychosocial burden generally increased with greater hair loss, we observed a notable trend in which these impacts declined among patients reporting complete baldness. This inflection point may reflect emotional exhaustion or treatment disengagement, marking a transition not previously described in the literature. Distress may peak before this shift, after which patients move from active management to reluctant acceptance, representing a critical window for intervention to preserve QoL and sustain engagement before motivation wanes. However, the small sample of respondents (n = 8) limits definitive conclusions. In addition, hair loss severity was categorized using broad percentage brackets, including a single ≥51% category, thereby limiting our ability to assess more granular trends at the most advanced stages of hair loss before complete baldness. Further research with finer stratification of hair loss severity is needed.

In addition, over 60% of CAPAIR respondents reported using nonevidence-based therapies, underscoring the desperation that can arise when patients perceive inadequate guidance or diminished hope.^9^ Similarly, Cook et al.^27^ found that many women with FFA were willing to spend up to their entire life savings in pursuit of a cure, highlighting the urgent need for timely intervention as well as the financial strain that can complicate management and potentially lead to disengagement. Narrative accounts have described clinicians minimizing scarring alopecia as cosmetic, contributing to poor communication and limited support, while others reported insurance denials based on similar misconceptions.^17^ One case described an insurance denial for a high-quality wig in a CCCA patient, despite clear benefits to her mood and functioning.^20^ Ingrassia et al.^39^ likewise reported that most patients lacked coverage for camouflaging agents, with 83% reporting financial stress. In our study, 37% of CCCA and LPP/FFA respondents reported spending >$100/month on treatment, and 18% in each group spent >$1,000/year on concealment tools. Moreover, over one-third of respondents incurred travel-related expenses to see specialists, including a subset who spent >$1,000/year, illustrating how limited access to knowledgeable care further compounds this burden. Indeed, 1 study found that many women with CCCA were initially evaluated by nondermatologists who failed to examine the scalp, while even among dermatologists, some reported a limited understanding of Black hair care.^5^ Similarly, UK dermatologists reported feeling unprepared to manage PCA because of inadequate training.^7^ Particularly in a disease with few effective therapies, these findings underscore the need for improved physician awareness and culturally competent training to facilitate earlier diagnosis, initiate treatment, and mitigate the distress patients experience when feeling dismissed or mismanaged.^5–7,15^

This study has several limitations inherent to its cross-sectional, self-reported survey design. Diagnoses, severity, and burden were participant-reported and not independently confirmed, raising the possibility of misclassification, inaccuracy, and recall bias. The anonymous format prevented response linkage, and item nonresponse limited internal consistency and data completeness. Subtype imbalances may also affect comparisons across PCA types. Recruitment primarily through the SAF may have introduced selection bias toward more engaged, informed, or severely affected individuals, limiting generalizability. In addition, potential survey dissemination via both email and social media (Facebook) precluded calculation of a true response rate, potentially further impacting selection bias. Despite these limitations, this large-scale, patient-reported study provides critical insight into an underrecognized and often misunderstood disease. PCA is not a cosmetic issue, but a chronic, multifaceted condition with tangible effects on physical comfort, daily functioning, finances, and psychological well-being. As existing research remains limited, characterizing lived experience in large, diverse cohorts is a necessary step toward improving awareness, care equity, and clinical guidance. Future research should continue to center on patient perspectives, define meaningful outcomes, and identify modifiable targets to improve the lives of those affected.

## Conflicts of interest

The authors made the following disclosures: J.T. has served as a consultant for AbbVie, Arcutis Biotherapeutics, Bristol-Meyers Squibb, Calliditas Therapeutics, Galderma, Johnson & Johnson, Leo Pharma, Novartis, Navigator Medicines, Primus Pharmaceuticals, Sanofi Genzyme, Stifel Financial, and UCB. He serves as an investigator for LEO Pharma, Priovant Therapeutics, and Sanofi. E.G.-Y. is an employee of Mount Sinai and has received research grants from and/or is a consultant for Abbvie, Arcutis, Almirall, Amgen, AnaptysBio, Apogee Therapeutics, Apollo Therapeutics Limited, Artax Biopharma Inc., Aslan, Astria, Bristol Meyers Squibb, Boerhinger-Ingelhiem, Calliditas, Cara Therapeutics, Celldex, Centrexion Therapeutics Corporation, Concert, Connect Biopharm, Coty, DBV Technologies, Eli Lilly, Enveda Biosciences, Escient Pharmaceuticals, Inc., Fairmount Funds Management LLC, FL2022-001, Inc., Galderma, Gate Bio, Google Ventures (GV), GSK Immunology, Incyte, Inmagene, Janssen Biotech, Jasper Therapeutics, Kymera Therapeutics, Kyowa Kirin, Leo Pharma, Matchpoint Therapeutics, Merck, Nektar Therapeutics, Novartis Pharmaceuticals, NUMAB Therapeutics AG, Nuvig, OrbiMed Advisors LLC, OTSUKA, Pfizer, Pharmaxis Ltd, Pioneering Medicine VII, Inc., Proteologix US Inc, Q32Bio, RAPT, RayThera, Inc, Regeneron Pharmaceuticals, RibonTherapeutics, Inc., Rocatinlimab, SAGIMET Bioscieces, Sanofi, SATO, Schrödinger, Inc., Sitryx, Sun Pharma Advanced Research Company (SPARC), Takeda, Teva Branded Pharmaceutical Products R&D, Inc, TRex, UCB, Ventyx Biosyences. B.U. is an employee of Mount Sinai and has received research funds (grants paid to the institution) from Bristol Myers Squibb, Incyte, Rapt Therapeutics, Pfizer, and Sanofi. He is also a consultant for AbbVie, Arcutis Biotherapeutics, Bristol Myers Squibb, Botanix Pharmaceuticals, Castle Biosciences, Ebla Holdco, Fresenius Kabi, Galderma, J&J, Leo Pharma, Lilly, Pfizer, Primus Pharmaceuticals, Sanofi, Sun Pharma, UCB, Veradermics, VRG Therapeutics. The other authors have no conflicts of interest.

## Funding

The CAPAIR survey was partially funded by Pfizer. No other funds, grants, or financial support were received.

## Study approval

Reviewed and approved by Mount Sinai IRB; STUDY-24-01578.

## Author contributions

JL and RO: Investigation, formal data analysis, writing—original draft, and writing—review and editing. JRP, ISJ, and MS: Conceptualization, project administration, funding acquisition, and writing—review and editing. JT, EGY, and BU: Conceptualization, investigation, formal data analysis, supervision, and writing—review and editing.

## Supplementary data

Supplementary material associated with this article can be found at https://links.lww.com/IJWD/A91, https://links.lww.com/IJWD/A92, https://links.lww.com/IJWD/A93 and https://links.lww.com/IJWD/A94.

## Supplementary Material


