# Supplementary material for: Multidimensional burden of scarring alopecia in women: findings from the CAPAIR study
Source: Int J Womens Dermatol. 2026 Jul 2;12(3):e268. doi: 10.1097/JW9.0000000000000268 (PMC13331435; doi:10.1097/JW9.0000000000000268)
Supplement: Supplementary file 1 [file jw9-12-e268-s001.pdf]

SDC, Table 1. Survey responses on self-reported financial and lifestyle burden, stratified by subtype of primary cicatricial alopecia, including central centrifugal cicatricial alopecia (CCCA), lichen planopilaris (LPP), and frontal fibrosing alopecia (FFA).

Characteristic	N[a]	CCCA, N (%)	LPP/FFA, N (%)	Overall, N (%)
Spending on non-prescription products, fads, trendy or "silver bullet" treatments in the past year	494			
$0–100		54 (34%)	126 (37%)	180 (36%)
$101–500		50 (32%)	78 (23%)	128 (26%)
$501+		53 (34%)	133 (39%)	186 (38%)
Cost of treatments per month	494			
$0–100		99 (63%)	213 (63%)	312 (63%)
$101–500		50 (32%)	111 (33%)	161 (33%)
$501+		8 (5%)	13 (4%)	21 (4%)
Spending on non-medical items (i.e., wigs, toppers, scarfs, hats, etc.) per year	494			
$0–100		59 (38%)	191 (57%)	250 (51%)
$101–500		39 (25%)	61 (18%)	100 (20%)
$501+		59 (38%)	85 (25%)	144 (29%)
If travel to see hair specialist or dermatologist, cost per year	494			
$0–100		100 (64%)	220 (65%)	320 (65%)
$101–500		42 (27%)	76 (23%)	118 (24%)
$501+		15 (10%)	41 (12%)	56 (11%)
Hours missed from work because of health problems, during past seven days[b]	917			
0 hours		95 (89%)	195 (81%)	290 (84%)
1-5 hours		9 (8%)	37 (15%)	46 (13%)
6+ hours		3 (3%)	8 (3%)	11 (3%)
Hours missed from work because of any other reason (vacation, holidays, time off), during past seven days	917			
0 hours		90 (79%)	201 (80%)	291 (80%)
1-5 hours		20 (18%)	31 (12%)	51 (14%)
6+ hours		4 (4%)	19 (8%)	23 (6%)
Hours worked, during past seven days	917			
0 hours		99 (65%)	163 (50%)	262 (54%)
1-5 hours		49 (32%)	161 (49%)	210 (44%)
6+ hours		5 (3.3%)	5 (1.5%)	10 (2.1%)
Work productivity impairment due to health problems, during past seven days[b]: Mean (SD)	494	0.85 (1.86)	1.40 (2.27)	1.23 (2.16)

Daily activity impairment due to health problems, during past seven days[b]: Mean (SD)	494	1.09 (2.23)	1.64 (2.47)	1.47 (2.41)

[a]Sample sizes may vary because of unreported data, and percentages are calculated based on the complete data of the column.

[b]Patients were instructed to replace the terms “health problems,” with the terms “scarring alopecia.”
