# Supplementary material for: Multidimensional burden of scarring alopecia in women: findings from the CAPAIR study
Source: Int J Womens Dermatol. 2026 Jul 2;12(3):e268. doi: 10.1097/JW9.0000000000000268 (PMC13331435; doi:10.1097/JW9.0000000000000268)
Supplement: Supplementary file 2 [file jw9-12-e268-s002.pdf]

SDC, Table 2. Physical symptoms and financial and lifestyle burden among respondents, stratified by extent of scalp hair loss.

Characteristic	N[a]	Less than 10%	11-25%	25-50%	51% or more	Bald
Current Symptoms	844					
Never/Rarely		133 (59%)	136 (40%)	50 (27%)	22 (25%)	3 (43%)
Sometimes/Always		91 (41%)	204 (60%)	135 (73%)	66 (75%)	4 (57%)
Blistering	910					
Never/Rarely		228 (95%)	347 (94%)	181 (91%)	89 (94%)	8 (100%)
Sometimes/Always		11 (5%)	23 (6%)	17 (9%)	6 (6%)	0 (0%)
Burning	844					
Never/Rarely		175 (78%)	260 (75%)	121 (66%)	53 (67%)	6 (75%)
Sometimes/Always		49 (22%)	89 (25%)	63 (34%)	26 (33%)	2 (25%)
Dryness, Crusting, Scales	824					
Never/Rarely		163 (72%)	227 (69%)	113 (64%)	68 (78%)	6 (75%)
Sometimes/Always		63 (28%)	100 (31%)	63 (36%)	19 (22%)	2 (25%)
Itching	719					
Never/Rarely		85 (43%)	103 (34%)	48 (34%)	21 (29%)	5 (63%)
Sometimes/Always		113 (57%)	197 (66%)	93 (66%)	51 (71%)	3 (38%)
Pain/Tenderness	804					
Never/Rarely		138 (64%)	185 (57%)	84 (48%)	35 (43%)	7 (100%)
Sometimes/Always		77 (36%)	141 (43%)	91 (52%)	46 (57%)	0 (0%)
Thinning Eyebrows	821					
Never/Rarely		94 (44%)	144 (43%)	84 (47%)	33 (39%)	4 (57%)
Sometimes/Always		121 (56%)	192 (57%)	94 (53%)	52 (61%)	3 (43%)
Thinning Hair	732					
Never/Rarely		38 (20%)	25 (9%)	14 (9%)	9 (10%)	1 (13%)
Sometimes/Always		156 (80%)	256 (91%)	148 (91%)	78 (90%)	7 (88%)
Spending on non-prescription products, fads, trendy or "silver bullet" treatments in the past year	494					
$0–100		58 (45%)	67 (36%)	33 (28%)	18 (32%)	4 (100%)
$101–500		35 (27%)	51 (28%)	29 (24%)	13 (23%)	0 (0%)
$501+		35 (27%)	67 (36%)	58 (48%)	26 (46%)	0 (0%)
Cost of treatments per month	494					
$0–100		87 (68%)	117 (63%)	72 (60%)	33 (58%)	3 (75%)
$101–500		36 (28%)	59 (32%)	43 (36%)	22 (39%)	1 (25%)
$501+		5 (4%)	9 (5%)	5 (4%)	2 (4%)	0 (0%)

Spending on non-medical items (i.e., wigs, toppers, scarfs, hats, etc.) per year	494					
$0–100		87 (68%)	103 (56%)	46 (38%)	12 (21%)	2 (50%)
$101–500		24 (19%)	38 (21%)	30 (25%)	8 (14%)	0 (0%)
$501+		17 (13%)	44 (24%)	44 (37%)	37 (65%)	2 (50%)
If travel to see hair specialist or dermatologist, cost per year	494					
$0–100		87 (68%)	119 (64%)	75 (62%)	37 (65%)	2 (50%)
$101–500		30 (23%)	45 (24%)	30 (25%)	11 (19%)	2 (50%)
$501+		11 (9%)	21 (11%)	15 (13%)	9 (16%)	0 (0%)
Hours missed from work because of health problems, during past seven days[b]	917					
0 hours		80 (90%)	116 (85%)	60 (72%)	33 (87%)	1 (100%)
1-5 hours		8 (9%)	17 (13%)	18 (22%)	3 (8%)	0 (0%)
6+ hours		1 (1%)	3 (2%)	5 (6%)	2 (5%)	0 (0%)
Hours missed from work because of any other reason (vacation, holidays, time off), during past seven days	917					
0 hours		81 (84%)	105 (72%)	70 (83%)	35 (88%)	0 (0%)
1-5 hours		12 (13%)	27 (19%)	10 (12%)	2 (5%)	0 (0%)
6+ hours		3 (3%)	13 (9%)	4 (5%)	3 (8%)	0 (0%)
Hours worked, during past seven days	917					
0 hours		71 (56%)	99 (54%)	60 (52%)	31 (56%)	1 (33%)
1-5 hours		53 (42%)	80 (44%)	53 (46%)	23 (42%)	1 (33%)
6+ hours		3 (2.4%)	3 (1.6%)	2 (1.7%)	1 (1.8%)	1 (33%)
Work productivity impairment due to health problems, during past seven days[b]: Mean (SD)	917	0.72 (1.48)	1.12 (2.03)	1.43 (2.42)	2.32 (2.87)	0.50 (1.00)
Daily activity impairment due to health problems, during past seven days[b]: Mean (SD)	917	0.73 (1.57)	1.38 (2.38)	1.81 (2.50)	2.68 (3.16)	1.25 (2.50)

[a]Sample sizes may vary because of unreported data, and percentages are calculated based on the complete data of the column.

[b]Patients were instructed to replace the terms “health problems,” with the terms “scarring alopecia.”
