# Supplementary material for: Multidimensional burden of scarring alopecia in women: findings from the CAPAIR study
Source: Int J Womens Dermatol. 2026 Jul 2;12(3):e268. doi: 10.1097/JW9.0000000000000268 (PMC13331435; doi:10.1097/JW9.0000000000000268)
Supplement: Supplementary file 3 [file jw9-12-e268-s003.pdf]

SDC, Table 3. Financial and lifestyle burden reported by patients, stratified by severity of self-reported scalp burning.

Characteristic	N[a]	Less Severe N=615	More Severe N=229	p-value
Spending on non-prescription products, fads, trendy or "silver bullet" treatments in the past year	453			0.429
Less than $100		122 (37%)	47 (37%)	
$101-$500		91 (28%)	25 (20%)	
$501-$1000		44 (13%)	25 (20%)	
$1001-$2500		44 (13%)	18 (14%)	
$2501-$5000		20 (6.1%)	8 (6.3%)	
$5000+		6 (1.8%)	3 (2.4%)	
Cost of treatments per month	453			0.008
0		37 (11%)	13 (10%)	
$1-100		183 (56%)	51 (40%)	
$101-250		70 (21%)	37 (29%)	
$251-500		25 (7.6%)	18 (14%)	
$501-1000		10 (3.1%)	3 (2.4%)	
$1001+		2 (0.6%)	4 (3.2%)	
Spending on non-medical items (i.e., wigs, toppers, scarfs, hats, etc.) per year	453			0.411
0		76 (23%)	41 (33%)	
$1-100		85 (26%)	26 (21%)	
$101-250		38 (12%)	11 (8.7%)	
$251-500		32 (9.8%)	13 (10%)	
$501-1000		39 (12%)	13 (10%)	
$1001+		57 (17%)	22 (17%)	
If travel to see hair specialist or dermatologist, cost per year	453			0.041
Less than $50		169 (52%)	55 (44%)	
$51-100		52 (16%)	13 (10%)	
$101-300		49 (15%)	30 (24%)	
$301-500		19 (5.8%)	14 (11%)	
$501-1000		19 (5.8%)	5 (4.0%)	
$1000+		19 (5.8%)	9 (7.1%)	
Hours missed from work because of health problems, during past seven days[b]	844			0.194
0 hours		193 (87%)	71 (77%)	
1-2 hours		8 (3.6%)	8 (8.7%)	
1-3 hours		9 (4.0%)	7 (7.6%)	
4-5 hours		6 (2.7%)	2 (2.2%)	
6-8 hours		7 (3.1%)	4 (4.3%)	
Other/Unknown		392	137	

Hours missed from work because of any other reason (vacation, holidays, time off), during past seven days	844			0.448
0 hours		192 (81%)	74 (78%)	
1-2 hours		13 (5.5%)	9 (9.5%)	
1-3 hours		13 (5.5%)	2 (2.1%)	
4-5 hours		5 (2.1%)	4 (4.2%)	
6-8 hours		13 (5.5%)	6 (6.3%)	
9-16 hours		1 (0.4%)	0 (0.0%)	
Other/Unknown		378	134	
Hours worked, during past seven days	844			0.587
0 hours		178 (56%)	70 (56%)	
1-5 hours		132 (42%)	55 (44%)	
6-10 hours		7 (2.2%)	1 (0.8%)	
Other/Unknown		298	103	
Work productivity impairment due to health problems, during past seven days[b]	453			0.004
Mean (SD)		0.97 (1.92)	1.73 (2.66)	
Daily activity impairment due to health problems, during past seven days[b]	453			0.024
Mean (SD)		1.24 (2.19)	1.94 (2.77)	

[a]Sample sizes may vary because of unreported data, and percentages are calculated based on the complete data of the column.

[b]Patients were instructed to replace the terms “health problems,” with the terms “scarring alopecia.”
