# Supplementary material for: Multidimensional burden of scarring alopecia in women: findings from the CAPAIR study
Source: Int J Womens Dermatol. 2026 Jul 2;12(3):e268. doi: 10.1097/JW9.0000000000000268 (PMC13331435; doi:10.1097/JW9.0000000000000268)
Supplement: Supplementary file 4 [file jw9-12-e268-s004.pdf]

SDC, Table 4. Financial and lifestyle burden reported by patients, stratified by severity of self-reported scalp pain/tenderness

Characteristic	N[a]	Less Severe N=449	More Severe N=355	p-value
Spending on non-prescription products, fads, trendy or "silver bullet" treatments in the past year	440			0.883
Less than $100		87 (36%)	78 (39%)	
$101-$500		67 (28%)	50 (25%)	
$501-$1000		38 (16%)	26 (13%)	
$1001-$2500		33 (14%)	26 (13%)	
$2501-$5000		12 (5.0%)	12 (6.1%)	
$5000+		5 (2.1%)	6 (3.0%)	
Cost of treatments per month	440			0.034
0		23 (9.5%)	27 (14%)	
$1-100		144 (60%)	89 (45%)	
$101-250		46 (19%)	56 (28%)	
$251-500		18 (7.4%)	20 (10%)	
$501-1000		9 (3.7%)	4 (2.0%)	
$1001+		2 (0.8%)	2 (1.0%)	
Spending on non-medical items (i.e., wigs, toppers, scarfs, hats, etc.) per year	440			0.585
0		59 (24%)	56 (28%)	
$1-100		64 (26%)	43 (22%)	
$101-250		26 (11%)	21 (11%)	
$251-500		23 (9.5%)	22 (11%)	
$501-1000		23 (9.5%)	25 (13%)	
$1001+		47 (19%)	31 (16%)	
If travel to see hair specialist or dermatologist, cost per year	440			0.047
Less than $50		123 (51%)	99 (50%)	
$51-100		33 (14%)	32 (16%)	
$101-300		41 (17%)	36 (18%)	
$301-500		11 (4.5%)	18 (9.1%)	
$501-1000		18 (7.4%)	4 (2.0%)	
$1000+		16 (6.6%)	9 (4.5%)	
Hours missed from work because of health problems, during past seven days[b]	804			0.55
0 hours		146 (88%)	112 (82%)	
1-2 hours		7 (4.2%)	9 (6.6%)	
1-3 hours		6 (3.6%)	9 (6.6%)	
4-5 hours		2 (1.2%)	4 (2.9%)	
6-8 hours		5 (3.0%)	3 (2.2%)	
Other/Unknown		283	218	

Hours missed from work because of any other reason (vacation, holidays, time off), during past seven days	804			0.407
0 hours		136 (79%)	115 (79%)	
1-2 hours		13 (7.5%)	9 (6.2%)	
1-3 hours		11 (6.4%)	4 (2.7%)	
4-5 hours		3 (1.7%)	6 (4.1%)	
6-8 hours		10 (5.8%)	11 (7.5%)	
9-16 hours		0 (0.0%)	1 (0.7%)	
Other/Unknown		276	209	
Hours worked, during past seven days	804			0.904
0 hours		127 (54%)	107 (55%)	
1-5 hours		103 (44%)	84 (43%)	
6-10 hours		5 (2.1%)	4 (2.1%)	
Other/Unknown		214	160	
Work productivity impairment due to health problems, during past seven days[b]	440			0.003
Mean (SD)		0.86 (1.78)	1.41 (2.33)	
Daily activity impairment due to health problems, during past seven days[b]	440			0.068
Mean (SD)		1.16 (2.10)	1.60 (2.53)	

[a]Sample sizes may vary because of unreported data, and percentages are calculated based on the complete data of the column.

[b]Patients were instructed to replace the terms “health problems,” with the terms “scarring alopecia.”
